# Waking inactivity as a welfare indicator in laboratory mice: investigating postures, facial expressions and depression-like states

**DOI:** 10.1098/rsos.221083

**Published:** 2022-11-02

**Authors:** Aileen MacLellan, Basma Nazal, Lauren Young, Georgia Mason

**Affiliations:** ^1^ Department of Integrative Biology, University of Guelph, 50 Stone Road East, Guelph, ON, Canada N1G 2W1; ^2^ Formerly Department of Animal Biosciences, University of Guelph, 50 Stone Road East, Guelph, ON, Canada N1G 2W1

**Keywords:** laboratory mice, welfare, inactivity, depression, posture, enrichment

## Abstract

Animal welfare assessment relies on valid and practical indicators of affect. In mice, the most widely used research vertebrates, lying still with eyes open, inactive-but-awake (IBA) in the home cage, has potential to be one such indicator. IBA is elevated in barren, conventional housing compared with well-resourced, enriched housing, and predicts immobility in Forced Swim Tests, a common measure of ‘helplessness’ in depression research. In Experiment 1, using females from three strains (C57BL/6, Balb/c and DBA/2), we first replicated past findings, confirming higher levels of IBA in conventional cages and a positive relationship between IBA and helplessness. We then extended this research to three other signs of depression: changes in weight and sleep, and reduced hippocampal volume. Here, IBA positively covaried with body mass index, with sleep in DBA/2s and conventionally housed BALB/cs, and negatively covaried with hippocampal volume in conventionally housed C57BL/6s. In Experiment 2, we sought to refine the phenotype of IBA to improve its accuracy as a welfare indicator. Here, scoring IBA performed in hunched postures appeared to improve its accuracy as an indicator in Balb/c mice. Additional research is now needed to further refine the phenotype of IBA and to confirm whether it reflects states consistent with depression, or instead other underlying poor welfare conditions.

## 1. Introduction

Identifying indicators of animal affect is essential for objectively assessing and improving welfare in the millions of animals kept in laboratory, farm and zoo settings. The most useful indicators are those that are well-validated as sensitive and specific to changes in affective state, in addition to being practical and non-invasive to implement [1,2]. Across species and contexts, such simple, well-validated indicators include repetitive behaviours like stereotypic pacing [3] or fur-plucking [4]. These are disturbing, clearly abnormal and widely recognized as indicators of poor welfare. But other, less active responses are now attracting attention and concern [5,6]. For example, the hunched postures of research primates isolated in metal indoor cages [7,8], the excessive time spent hiding by wild-caught striped mice brought into the laboratory [9], and the ‘flat necked’ postures of unresponsive, anhedonic horses [10,11] have become recognized as signs of poor wellbeing. For laboratory mice (*Mus musculus*), the most widely used vertebrates in research [12,13], welfare assessment often relies on clinical signs (e.g. coat and body condition, hunched postures) and overtly abnormal active behaviours like stereotypic route-tracing or bar-mouthing. However, recent research has shown that some mice demonstrate more subtle, inactive responses to suboptimal conditions. Here, some individuals spend excessive time simply lying motionless, but with their eyes are open (indicating that they are awake) during the dark, active phase of their circadian rhythm. This ‘inactive-but-awake’ (IBA) behaviour reliably occurs at higher levels in conventional ‘shoebox’ cages than in well-resourced, environmentally enriched housing [14–17] (the label ‘well-resourced’ being applied hereafter to describe complex environments, since the more common term ‘enriched’ implies conditions do more than meet basic needs; see [18,19,20] for further discussion). Such housing effects provide construct validity for the use of IBA as an indicator of negative affect, since it is well established that these barren conditions (e.g. containing only bedding and nesting material) are aversive for mice: they restrict highly motivated, species-typical behaviours (e.g. hiding, foraging, exploring [21,22]), and individuals kept in conventional cages will push heavy weights to leave them and access well-resourced environments [23]. Moreover, conventional cages elevate anxiety [24], increase stereotypic behaviours (e.g. [25]) and compromise rodent health (increasing morbidity and mortality in a manner consistent with chronic stress [19]). IBA thus holds promise as a simple tool for welfare assessment.

Research investigating this form of inactivity has led to the suggestion that IBA may be a ‘depression-like’ response. Multiple lines of reasoning support this claim. First, in humans, chronic stress and suboptimal conditions are risk factors for clinical depression [26–28], and second, clinical depression can involve ‘psychomotor retardation’ that manifests as reduced activity [29]. Third, mice demonstrating high levels of IBA can be less sociable [30], in line with the way that depressed humans often interpret social interactions as unrewarding [31,32]. Fourth, in mice, high levels of IBA have been shown to predict high levels of immobile floating (instead of active swimming and escape attempts) during Forced Swim Tests [14]. This is a measure of ‘helplessness’ common in biomedical research using rodents to model depression [33,34]. These tests aim to approximate the ways that clinically depressed humans can have passive responses to adversity (due to beliefs that desired outcomes are unlikely, aversive outcomes are likely, and one's actions have no impact on such consequences [35,36]). Finally, recent research has also shown that IBA is reduced by an antidepressant (venlafaxine [17]).

Building on this, our research aims here were twofold. The first was to further test the hypothesis that IBA reflects a housing-induced depression-like state in mice (Experiment 1). The second was to refine the way IBA is identified, to better distinguish between it and normal forms of inactivity, thus improving IBA's accuracy as a welfare indicator (Experiment 2). The first aim is important because, while intriguing, the evidence to date is not sufficient to confirm that IBA mice are in states akin to depression. For one, a confirmed diagnosis of depression requires the co-occurrence of at least *five* signs or symptoms (out of nine: low mood, anhedonia, weight loss or gain, insomnia or hypersomnia, psychomotor agitation or retardation, fatigue or loss of energy, feelings of worthlessness or guilt, inability to think or concentrate, and recurrent thought of death or suicide) [26]. In addition, helplessness is *not* one of these required signs, and, despite its ubiquitous use in pre-clinical research, the construct and face validity of rodent Forced Swim Tests have also been called into question [37–39]. Furthermore, response to anti-depressants is also not proof of depression: antidepressant drugs can treat other conditions too (e.g. anxiety [40] and pain [41]), and yet also commonly fail to alleviate depression in human populations [42–44]. In Experiment 1, we therefore aimed to not only replicate past Forced Swim Test effects, but knowing the limitations of these tests, to also assess two diagnostic criteria for depression: changes in weight and sleep [26]. We also assessed a biomarker of the disorder (one not used in clinical diagnosis, but very often apparent in imaging studies): reduced hippocampal volume [45,46].

Experiment 2 had a complementary aim: to improve the accuracy of IBA as an indicator of poor welfare. Current ethogram descriptions of IBA only require that a mouse remains motionless with eyes open for 3 seconds or more [14,15,17]. Despite being simple and objective, this broad description probably leads to the classification of diverse forms of inactivity as IBA, including normal resting behaviour. This may be why mice in well-resourced housing can be scored as performing it (albeit at low levels [14,15]). After all, inactivity *is* heterogeneous: it may occur in diverse negative states (not only depression, but also sickness, boredom, fear and others [5]); but it also occurs during neutral and positive ones (e.g. while resting or sun basking [47]). In humans, and some other species (e.g. mink [48]), such differing forms often diverge in appearance, potentially allowing them to be distinguished in ethograms. We therefore investigated whether differences in IBA between conventional and well-resourced housing conditions are not only quantitative but also qualitative, the IBA of conventionally housed mice diverging in appearance from similar behaviour in well-resourced animals. We focused on three features that could readily be scored cageside. The first was posture: rodents show hunched postures when defeated by aggressors [49] and when sick [50,51], suggesting this could be a broad sign of negative affect. Inactive postures in stressed non-human primates [8,52] and depressed humans [53] likewise often involve hunched backs. The second was partially closed ‘squinting’ eyes. In mice, this occurs during pain [54,55] and also during fear [56], suggesting a broad link with negative affect. The third was holding the ears back. Again, in mice, this occurs during pain (being part of the Mouse Grimace Scale) [54], but in horses, this also helps characterize the abnormal inactivity of anhedonic, ‘withdrawn’ individuals [11]. We therefore investigated whether any of these aspects of expression or posture would distinguish between the IBA of mice in high- and low-welfare housing conditions, so helping to refine the phenotype of forms that specifically indicate negative affect.

## 2. Material and methods

### 2.1. Experiment 1: inactive-but-awake behaviour and depressive signs

#### 2.1.1. Animals and housing

Subjects were 90 female mice from three strains (30 C57BL/6NCrl, 30 Balb/cAnNCrl and 30 DBA/2NCrl, henceforth ‘C57’, ‘Balb’ and ‘DBA’, respectively) purchased from Charles River Laboratories (Raleigh, North Carolina, USA). Female mice were used throughout all experiments reported here to allow concurrent group housing of this social species with environmental enrichment, yet without resource guarding and aggression that can arise in some strains of male mice [57]. Mice were selected from a population of 165 animals (55 C57s, 55 Balbs and 55 DBAs) that had arrived at the facility at three or four weeks of age and were randomly assigned to well-resourced (*N* = 22) or conventional (*N* = 33) cages. Mice were reared to adulthood in mixed-strain trios with one C57, one Balb and one DBA per cage. This housing system facilitates investigation of IBA in three phenotypically distinct mouse strains: C57s, a strain prone to inactivity and helplessness in response to stressors; DBAs, a strain that instead demonstrates hyperactivity and stereotypic behaviour; and Balbs, a strain that displays moderate levels of both behavioural phenotypes [15,58]. Yet importantly, mixed-strain housing also maintains welfare-relevant behavioural phenotypes (compared with same-strain housing); increases statistical power through the use of a split-plot design and reducing data variability; and allows for individual identification without aversive marking methods [59]. Individuals included in this experiment had not had any cagemates removed from their home cage, due to health concerns or whisker barbering, by 16 months of age when Forced Swim Tests were conducted (i.e. cages with two individuals were excluded to ensure equivalent social and housing density experience for all experimental animals).

**Figure 1.**
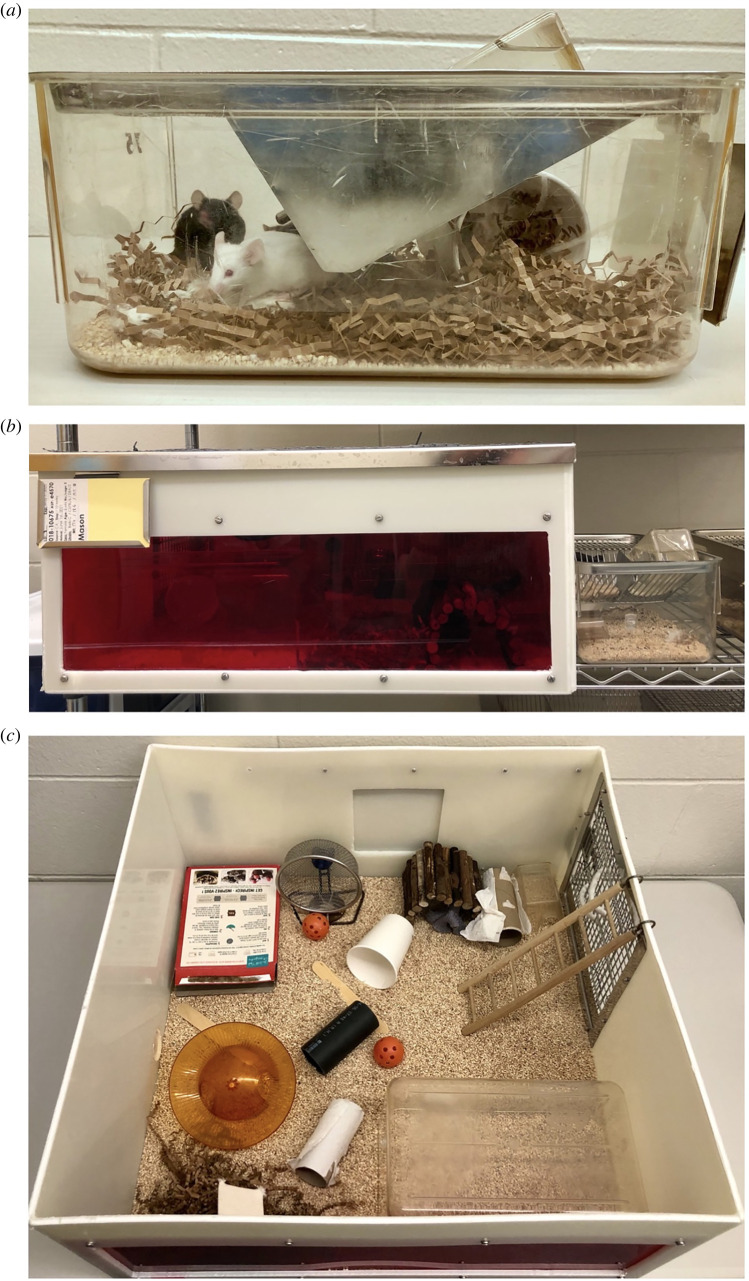
(*a*) Conventional cage (note that paper cup was not included in Experiment 1), (*b*) front view of well-resourced cage with attached annex cage on the right-hand side (as outlined in Experiment 2), (*c*) overhead view of well-resourced cage with biologically relevant enrichments. A multi-level environment was also created by hanging enrichments like sock hammocks and egg cartons from cage lids (not shown here). Note: image (*a*) is not to scale with images (*b*) and (*c*).

Conventional housing comprised transparent polyethylene cages (27 cm length **×** 16 cm width **×** 12 cm height, Allentown Inc.), containing corn cob bedding and two types of nesting material (crinkled paper strips and cotton nestlets). Well-resourced cages were opaque plastic, with the exception of one transparent red plastic wall to allow observers to see into the cage during behavioural observations (60 cm l **×** 60 cm w **×** 30 cm h) (figure 1*a*). Mice in well-resourced housing were provided with biologically relevant enrichments, as described by Nip *et al*. [15], which they are highly motivated to access [23] (figure 1*b*,*c*). Conventional and well-resourced cages were evenly distributed on racks to control for differences in light exposure [22]. The colony room was maintained at 23 ± 1°C and 35–55% relative humidity, on a reverse 12 : 12 h light cycle, with lights off at 07.00 and lights on at 19.00. Food (Harlan Teklad 14% protein rodent chow) and tap water were available ad libitum. Handling of mice during all procedures followed either tunnel or hand cupping methods, in order to minimize aversive effects of handling [60].

#### 2.1.2. Behavioural observations

Home cage behavioural data were collected via live scan sampling. Observations were carried out for 4 h by two concordant observers (Cohen's *κ* ≥ 0.70), starting 2 hours after lights off at 09.00 [61,62]. Observers moved from cage to cage identifying and recording the first observed behaviour of each mouse according to the ethogram in table 1. Each cage was scanned approximately every 10 min. Observation periods lasted 4–6 days and were repeated when mice were three, five, eight, 10 and 14 months of age. To account for the fact that mice from well-resourced cages had more opportunities to be out of sight, each behaviour was calculated as a proportion of visible scans. For all behaviours used in statistical analyses, a lifetime average was calculated from the five observation periods.

**Table 1.** Ethogram of relevant behaviours used during live observations. For extended ethogram see Nip *et al*. [15].

category	behaviour	description
inactivity	IBA	mouse is motionless, with eyes open for at least 3 s
	resting	mouse is still with eyes out of sight from the observer
	sleeping	mouse is still with eyes closed for at least 3 s
stereotypic behaviours	bar-mouthing	mouse holds cage bar in diastema and sham-bites for 3 or more seconds
	back-flipping	mouse throws body in backward arch off cage floor or wall and lands in the initial body orientation for three or more repetitions
	twirling	mouse hangs by its forepaws on cage lid and turns body in tight circles for three or more repetitions
	patterned lid-climbing	mouse walks/runs on cage lid using all four paws in a fixed, idiosyncratic route for three or more repetitions
	route-tracing	mouse walks/runs on cage floor in a fixed idiosyncratic route, for three or more repetitions
	combinations	combinations of two or more stereotypic behaviours (e.g. route-tracing with back-flipping)
other	out of sight active	specific activity is obscured from observer's view, but mouse is clearly active
	out of sight	mouse is completely out of the observer's view. It is unknown whether they are active or not
	other	behaviours not listed here

Levels of both IBA and sleep were assessed during these observations with IBA being classed as ‘[a mouse that is] motionless with eyes open for at least 3 s’, and sleep identified as ‘still with eyes closed for at least 3 s’. It is important to note that while the gold standard for sleep assessment in mammals, including mice, is recording of electroencephalography (EEG) and electromyography, less invasive methods such as video analysis to assess sleep through lack of movement have been validated [63]. Therefore, live scan sampling is a relevant proxy for measuring this behaviour.

#### 2.1.3. Forced Swim Tests

Forced Swim Tests were conducted when mice were 16 months of age. Tests were conducted over 4 consecutive days in a test room adjacent to the colony room between 10.00 and 13.30 under white light. To reduce any risk of hypothermia for mice, the test room was kept at 29**°**C, and a space heater and heating pad were kept on hand. Home cages were brought from the colony room to the test room to allow mice to habituate in their home cage for 5 minutes prior to testing. Each cage was randomly assigned to a day of testing, counterbalancing across treatment. Mice within each cage were randomly assigned to one of three clear plastic test cylinders (22 cm h **×** 21 cm w) filled with 18 cm of gently warmed water (25.15°C **±** 0.45, 24.70–25.70°C). Cylinders were placed side by side and visually separated by opaque screens. All handling and monitoring of mice during testing was performed by a researcher blind to housing treatment and IBA status. During each Forced Swim Test, mice were lowered into water using a plastic tunnel and video recorded for 6 min (2 min for habituation, 4 min for testing). Mice were monitored closely by experimenters while in the water, with the aim of immediately retrieving any mouse whose nose submerged twice. However, no retrievals were required as all mice swam or floated successfully. When each test ended, each mouse was removed from the cylinder, towel dried and returned to their home cage. Between each test, cylinders were cleaned with disinfectant (Clorox™ hydrogen peroxide), rinsed and the water was replaced.

Mice were given dried sweetened banana chips and Honey Nut Cheerios™ once returned to their home cage, and monitored closely for signs of hypothermia. One cage of mice was placed on the heating pad as a precaution, and all cagemates recovered quickly with this aid. These mice were also checked by a technician before being returned to their cage. All cages remained in the test room for 20 min to allow mice to dry completely before being returned to the colony room. Test videos were scored using JWatcher™ 0.9 software by an observer blind to treatment and IBA status. Time spent swimming and floating immobile during the 4 min test period was assessed. Mice were scored immobile when they remained floating for a minimum of 2 s with at least three legs motionless [14,64].

#### 2.1.4. Body mass indices

Each mouse was weighed, hand restrained and held so a photo could be taken of the ventral side of her body. Using ImageJ software (1.52a, National Institutes of Health, USA), an observer blind to housing treatment and IBA status measured nose to anus length from photos, and body mass index (BMI) was calculated (BMI = weight in grams/[nose-to-anus length cm]^2^). These BMI values were thus used as a proxy for the depression criterion of weight loss or gain, since unusually high or low BMIs are characteristic of clinically depressed human populations [65], and BMI more accurately reflects high levels of body fat (indicative of weight gain) in mice by accounting for differences in body length.

#### 2.1.5. Hippocampal volume

A subset of 36 animals (12 conventional housed C57s, seven well-resourced C57s, 11 conventional housed DBAs and six well-resourced DBAs) underwent transcardial perfusion at 17 months of age to collect brains for hippocampal volume assessment. Balb mice were adopted out at this time point, but this allowed us to focus on the most extreme phenotypes of IBA (C57 mice being prone to particularly high levels of IBA, and DBA mice performing little of the behaviour; see Results). Mice were selected for perfusion by splitting individuals into quartiles based on IBA and stereotypic behaviour, and selecting equal numbers of animals from each quartile to generate a representative sample. Each mouse was anaesthetized with 150 mg kg^−1^ pentobarbital and transcardially perfused with saline followed by 4% paraformaldehyde. Brains were extracted, immersion fixed in 4% PFA then moved to sucrose solution until they sank, before being stored at −80°C. Whole brains were sectioned coronally at 50 µm via cryostat (Leica CM1950, Leica Biosystems) and divided into four full brain replicates. Cytochrome oxidase histochemistry was implemented for staining because it is a marker of neuronal activity [66] (and these brains were being used to simultaneously test hypotheses regarding the neurobiology of stereotypic behaviour in another experiment; see Kitchenham [67]), and also because pilot tests confirmed clear visibility of the hippocampus with this stain. Histochemistry was carried out using a modified version of Lauer *et al*.'s [66] protocol described in detail by Kitchenham [67].

Unfortunately, nine brains had to be excluded from hippocampal volume assessment due to breakage that occurred in the hindbrain during sectioning (large pieces breaking off making it impossible to estimate volume from their inconsistent/unknown thickness). This was caused by the ventricular enlargement in these old animals. The hippocampi of the remaining 27 brains (10 conventionally housed C57s, six well-resourced C57s, five conventionally housed DBAs, six well-resourced DBAs) were identified according to the Allen Mouse Brain Atlas [68], traced and area measured using ImageJ software (1.52a, National Institutes of Health, USA) by an observer blind to housing treatment and IBA status. Hippocampal volume was then calculated using Cavalieri's Principle (Total volume = Total area × Inverse of sampling fraction × Coronal section thickness).

#### 2.1.6. Statistical analyses

All data were analysed with general linear mixed models in SAS® 9.4 or JMP (v. 16, SAS Institute 2021). Transformations were performed where necessary to meet assumptions of normality and homogeneity of residuals (arcsine square root or Box-Cox transformations). Models investigating housing effects on IBA and Forced Swim Tests included the fixed effects of Housing, Strain and their two-way interaction along with Cage nested within Housing as a random effect. All Forced Swim Test models also included BMI and Water Temperature as covariates since these factors can influence motility and buoyancy [69,70]. Thus, models testing whether IBA predicted proportion of time spent immobile during Forced Swim Tests included the fixed effects of IBA, Housing, Strain and all possible interactions, as well as BMI and Water Temperature. Cage nested within Housing was also included as a random effect. Models investigating the relationship between IBA and levels of sleep, BMI and hippocampal volume included IBA, Housing, Strain and all possible interactions, with Cage nested within Housing as a random effect. Hippocampal volume models also included Total Brain Volume as a covariate to avoid confounding effects of overall brain size [71]. Only statistically significant (*p* < 0.05) or trend interactions (*p* < 0.1) with Housing or IBA were investigated, since these were crucial for testing our hypotheses (Tukey–Kramer adjustments for multiple comparisons being included during these investigations). Finally, effect sizes were calculated as Cohen's *d*. Effect sizes were considered small if Cohen's *d* was less than 0.5, medium if between 0.5 and 0.8, and large if 0.8 or above.

### 2.2. Experiment 2: refining the phenotyping of inactive-but-awake behaviour

#### 2.2.1. Animals and housing

Subjects were 44 C57, 56 Balb and 44 DBA mice from two cohorts purchased from Charles River Laboratories (Raleigh, North Carolina, USA). The first cohort consisted of 11 C57 and 13 Balb females who had been used in another experiment [72], and the second cohort included 33 C57, 43 Balb and 44 DBA females. Again, mice arrived at the facility at three or four weeks of age, were randomly assigned to well-resourced (Cohort 1 *N* = 9; Cohort 2 *N* = 22) or conventional cages (Cohort 1 *N* = 9, Cohort 2 *N* = 22) and reared to adulthood in mixed-strain housing. In Cohort 1, each cage contained four mice: one C57, one Balb and two DBAs (being used in a parallel experiment on the neurobiology of stereotypic behaviour), while Cohort 2 contained one mouse from each strain as described in the previous experiment [59]. All C57 and Balb mice from Cohort 1 and all mice from Cohort 2 who had not been removed at the time of behavioural observations (see §2.2.2. below) for barbering cagemate's whiskers, or other naturally occurring health issues were included in experiments. Housing and room conditions were the same as those described in Experiment 1, with two exceptions: conventional cages now contained a paper cup shelter (figure 1*a*) and well-resourced cages included an attached ‘annex’ cage which mice had free access to via a tunnel (figure 1*b*). These annex cages avoided aversive ‘chasing’ through complex environments [73] by facilitating catching and handling, since well-resourced mice were trained to enter this attachment when a cup full of Cheerios™ was shaken (as described by Resasco *et al*. [72]).

#### 2.2.2. Behavioural observations

**Figure 2.**
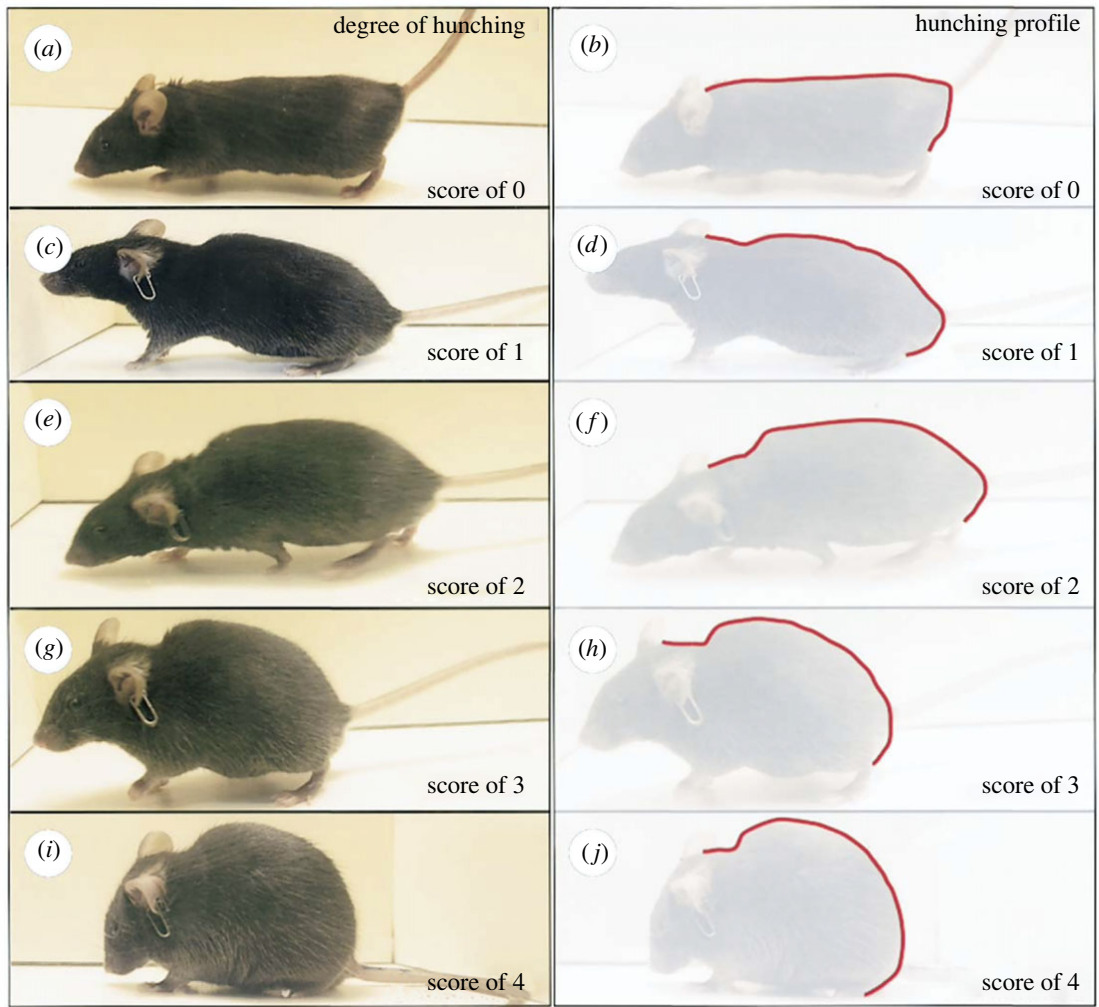
Hunched posture scoring system. All bouts of IBA performed with a back arch score of 1 and above were classed as ‘hunched’. Reprinted from *Gastroenterology, 131, Sevcik et al. [74] Endogenous opioids inhibit early-stage pancreatic pain in a mouse model of pancreatic cancer, 900–910, 2006,* with permission from Elsevier.

**Figure 3.**
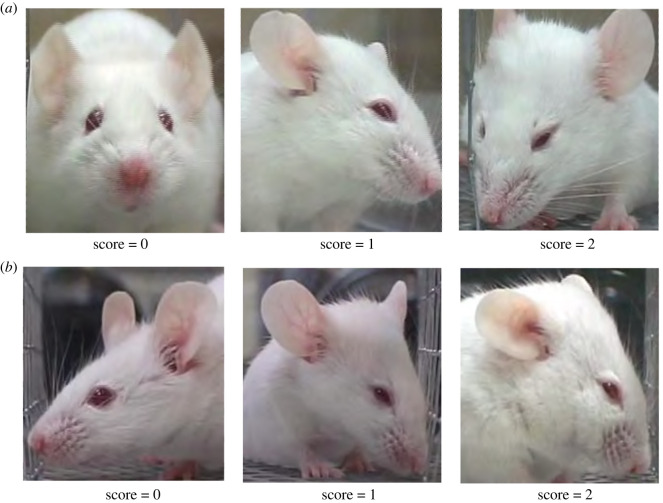
Images of mouse (*a*) eye squinting scores and (*b*) ear position scores. Reprinted from *Nature Methods, 7, Langford et al. [54] Coding of facial expressions of pain in the laboratory mouse, 447–449, 2010,* with permission from Springer Nature.

When mice were 17 months of age (Cohort 1) or 11 months of age (Cohort 2) home cage behavioural data were collected via live scan sampling following a modified version of the protocol described in Experiment 1 (the age discrepancy between the groups being a result of COVID-19 lockdowns that delayed research for Cohort 1). Again, live scan sampling observations were conducted for 4 h under red light between 09.00 and 13.00. COVID-19 room occupancy limits in place during Cohort 1 observations meant only one live observer could be present for data collection. Thus, during Cohort 1 observations, cages were scanned approximately every 20 min for 4 consecutive days. In Cohort 2, data were again collected by two concordant observers (Cohen's *κ* ≥ 0.70) scanning cages approximately every 10 min over 2 consecutive days (following recommendations from Adcock [62] for representative live sampling data), both protocols yielding approximately the same number of scans per mouse during the observation period. During each scan, the first behaviour of each mouse was recorded according to the ethogram in table 1. Whenever IBA was identified, the observer then noted the ear position, degree of eye squinting and posture/body position of the mouse (table 2). Hunched postures were identified according to descriptions in mouse welfare assessment schemes [51] and clinical scoring systems [74] (table 2; figure 2). Ear position and eye squinting were scored according to the Mouse Grimace Scale [54] (table 2; figure 3). Other components of the scale (e.g. nose or cheek bulge, changes in whisker position) were not assessed because too subtle for live detection under red light.

**Table 2.** Characteristics of IBA assessed with the aim of refining the phenotype. Hunched postures were identified according to descriptions provided by Spangenberg & Keeling's Appendix 1 [51] as well as images and descriptions outlined by Sevcik *et al*. [74] (figure 2). Ear position and eye squinting are based upon the NC3Rs Mouse Grimace Scale (figure 3).

category	classification	description
posture	hunched	Back is arched/rounded. This can include mild to severe rounding (i.e. scores of 1 and above according to Sevcik *et al*. [74]).
	not hunched	Back is not arched or hunched beyond the normal curvature of the spine (i.e. score of 0 according to Sevcik *et al*. [74]).
eye squinting	0	No narrowing of the eyes (orbital tightening) is detected.
	1	Eyes are slightly narrowed and orbital muscles around the eyes are contracted. Mouse must display a narrowing of the orbital area, a partially closed eyelid, or an eye squeeze. An eye squeeze is defined as the orbital muscles around the eyes being contracted. A wrinkle may be visible around the eye.
	2	Eyes are narrowed to less than half of their size when opened. Orbital muscles around the eyes are contracted.
ear position	0	Ears are directed forward and approximately perpendicular to the head.
	1	Ears are pulled slightly back, rotated partially outwards and the space between ears may appear wider.
	2	Ears are pulled back, approaching or laying back on the head. Ears are rotated outwards and the space between ears may appear wider.

#### 2.2.3. Statistical analyses

Data first were analysed with general linear mixed models as in Experiment 1. Here, models for all forms of IBA included the fixed effects of Housing, Strain and Cohort plus their two-way and three-way interaction, along with Cage nested within Housing as a random effect. However, data could not meet parametric assumptions, so the two cohorts were analysed separately. Models for Cohort 1 included the fixed effects of Housing, Strain and their two-way interaction along with Cage nested within Housing as a random effect. Statistically significant (*p* < 0.05) or trend Housing*Strain interactions (*p* < 0.1) were investigated, using Tukey–Kramer adjustments for multiple comparisons, since these were essential for testing our hypothesis. Cohen's *d* effect sizes were calculated and compared in order to determine which IBA characteristics were most specific to standard housing conditions. For Cohort 2, models for specific forms of IBA did not meet parametric assumptions (even after transformation). Therefore, housing effects were investigated using non-parametric Wilcoxon rank sum tests for each strain. To allow for comparison with total IBA, a Wilcoxon rank sum test was also conducted for total IBA in each strain in addition to the mixed model described above. Wilcoxon *r-*value effect sizes were calculated from Z statistics [75] and considered small if *r* was less than 0.3, medium if between 0.3 and 0.5, and large if 0.5 or above.

## 3. Results

### 3.1. Experiment 1: inactive-but-awake behaviour and depressive signs

#### 3.1.1. Replicating past housing and Forced Swim Test effects

**Figure 4.**
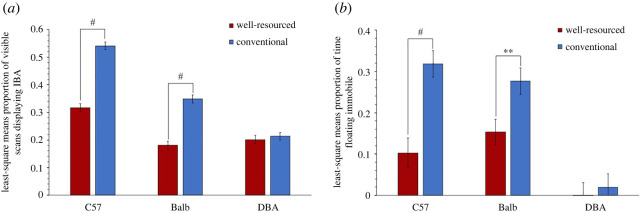
Housing effects on (*a*) home cage IBA least-square means ± s.e. (data arcsine square root transformed) and (*b*) time spent immobile during Forced Swim Tests least-square means ± s.e. (data arcsine square root transformed; * = *p* ≤ 0.05, ** = *p* ≤ 0.01, *** = *p* ≤ 0.001, ^#^ = *p* < 0.0001).

When investigating the effects of housing on IBA, a significant Housing*Strain interaction (*F*_2,56_ = 41.18, *p* < 0.0001) was detected. This was because the simple effect of Housing was significant in C57 (*t* = 10.99, d.f. = 71.47, *p* < 0.0001, Cohen's *d* = 4.024) and Balb mice (*t* = 8.23, d.f. = 71.47, *p* < 0.0001, Cohen's *d* = 3.013) (with animals showing more IBA in conventional housing), but not DBAs, who showed little IBA overall (*t* = 0.60, d.f. = 71.47, *p* = 0.552, Cohen's *d* = 0.219) (figure 4*a*, table 3). Similarly, models investigating housing effects during Forced Swim Tests detected a significant Housing*Strain interaction (*F*_2,55.64_ = 4.70, *p* = 0.013), revealing that the simple effects of Housing were significant in C57s (*t* = 4.96, d.f. = 81.54, *p* < 0.0001, Cohen's *d* = 0.164) and Balbs (*t* = 2.71, d.f. = 81.97, *p* = 0.009, Cohen's *d* = 0.994) (figure 4*b*) (with conventionally housed mice spending more time immobile), but again not DBA mice (*t* = 0.45, d.f. = 81.97, *p* = 0.657, Cohen's *d* = 0.162) who showed little Forced Swim Test floating.

**Table 3.** Summary of descriptive statistics for key variables in Experiment 1.

variable	housing	strain	*N*	mean	s.d.
proportion of visible scans displaying IBA	well-resourced	Balb	14	0.033	0.01
		C57	14	0.1	0.038
		DBA	14	0.042	0.023
	conventional	Balb	16	0.12	0.04
		C57	16	0.266	0.047
		DBA	16	0.048	0.027
proportion of visible scans displaying stereotypic behaviour	well-resourced	Balb	14	0.021	0.041
		C57	14	0.002	0.003
		DBA	14	0.013	0.007
	conventional	Balb	16	0.093	0.051
		C57	16	0.014	0.009
		DBA	16	0.238	0.091
proportion of visible scans sleeping	well-resourced	Balb	14	0.012	0.008
		C57	14	0.007	0.005
		DBA	14	0.014	0.009
	conventional	Balb	16	0.029	0.019
		C57	16	0.017	0.005
		DBA	16	0.01	0.01
proportion of time immobile during Forced Swim Tests	well-resourced	Balb	14	0.174	0.161
		C57	14	0.132	0.153
		DBA	14	0.001	0.002
	conventional	Balb	16	0.315	0.169
		C57	16	0.407	0.24
		DBA	16	0.008	0.026
BMI	well-resourced	Balb	14	0.276	0.054
		C57	14	0.346	0.086
		DBA	14	0.266	0.06
	conventional	Balb	16	0.212	0.014
		C57	16	0.322	0.068
		DBA	16	0.211	0.036
hippocampal volume (mm^3^)	well-resourced	C57	6	14.347	1.366
		DBA	6	14.180	1.308
	conventional	C57	10	13.966	1.198
		DBA	5	15.399	1.219

**Figure 5.**
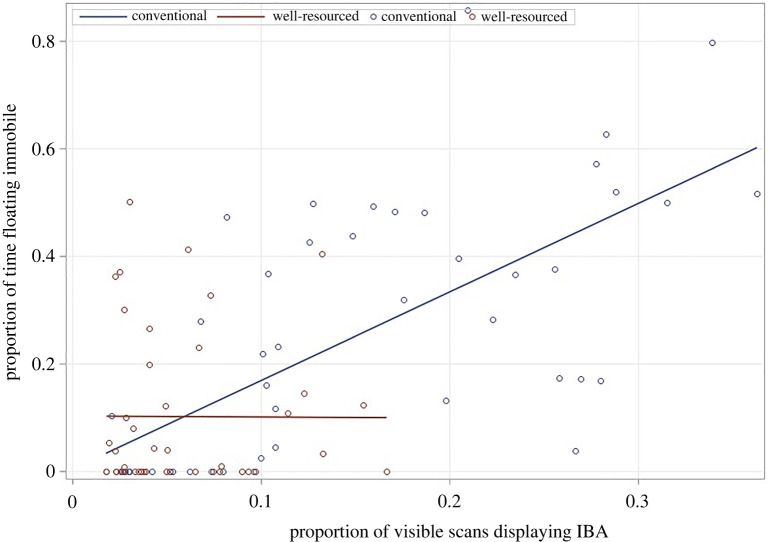
Relationship between proportion of visible observations where mice demonstrated IBA, and proportion of time spent immobile during Forced Swim Tests. Raw data are presented here for ease of interpretation, although analyses used arcsine square root transformation.

When assessing the relationship between IBA and Forced Swim Test floating, a significant IBA*Housing interaction was detected (*F*_1,53.81_ = 4.90, *p* = 0.031). Splitting by housing revealed that IBA predicted immobility during Forced Swim Tests in conventionally housed mice (*F*_1,23.4_ = 4.59, *p* = 0.043), with high IBA mice spending more time floating immobile. By contrast, this relationship was not significant for well-resourced animals, for whom, as mentioned, both IBA and floating were low (*F*_1,21.74_ = 1.80, *p* = 0.1937) (figure 5).

#### 3.1.2. Investigating additional signs of depression

**Figure 6.**
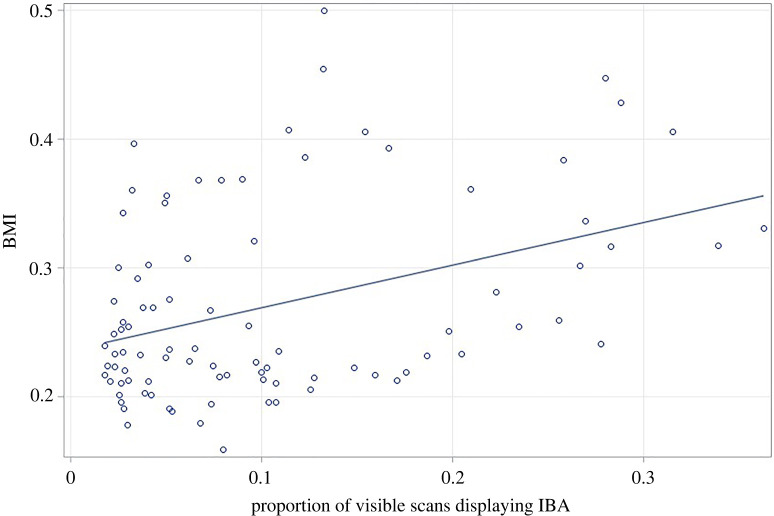
Relationship between the proportion of visible observations where mice demonstrated IBA, and BMI. Raw data are presented here for ease of interpretation, although analyses used logarithmic transformation.

When investigating the effects of housing on BMI, a significant Housing*Strain interaction (*F*_2,56_ = 3.22, *p* = 0.047) was detected. This was because the simple effect of Housing was significant in Balb (*t* = −3.57, d.f. = 66.18, *p* < 0.001, Cohen's *d* = −1.307) and DBA mice (*t* = −3.21, d.f. = 66.18, *p* = 0.002, Cohen's *d* = −1.173) (with well-resourced animals having greater BMIs), but not C57s (*t* = −0.93, d.f. = 66.18, *p* = 0.354, Cohen's *d* = −0.342). Assessing the relationship between BMI and IBA showed that mice spending more time IBA also had greater BMIs (*F*_1,77.9_ = 8.33, *p* = 0.005) (figure 6).

**Figure 7.**
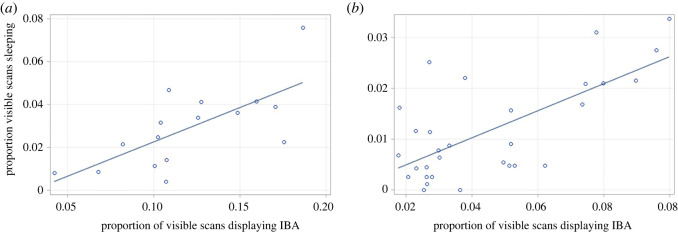
Relationship between proportion of visible observations where mice displayed IBA, and proportion of visible scans sleeping in (*a*) well-resourced Balb mice and (*b*) DBA mice.

Models investigating housing effects on sleep during the active phase revealed a significant Housing*Strain interaction (*F*_2,56_ = 11.84, *p* < 0.0001). This was because the simple effect of Housing was significant in C57 (*t* = 3.19, d.f. = 68.46, *p* = 0.002, Cohen's *d* = 1.168) and Balb mice (*t* = 3.76, d.f. = 68.46, *p* < 0.001, Cohen's *d* = 1.375) (with conventionally housed animals spending more time sleeping), but not DBAs (*t* = −1.35, d.f. = 68.46, *p* = 0.182, Cohen's *d* = −0.495). Investigating the relationship between IBA and sleep revealed a significant IBA*Strain interaction (*F*_2,63.95_ = 9.78, *p* < 0.001) and a trend for IBA*Housing (*F*_1,77.66_ = 3.78, *p* = 0.056). Splitting by housing revealed a significant IBA*Strain interaction in the conventional housing model (*F*_2,37.39_ = 10.89, *p* < 0.001), and when split again by strain a positive relationship between IBA and sleep during the active phase was also detected for conventionally housed Balbs (*F*_1,14_ = 13.05, *p* = 0.003) but not other strains nor well-resourced mice (figure 7*a*). Splitting data instead by strain, surprisingly there were no interactive effects of Housing, nor any significant relationship between IBA and sleep found in models for C57 (*F*_1,26_ = 0.04, *p* = 0.848) or Balb mice (*F*_1,26_ = 0.80, *p* = 0.380), but unexpectedly for DBAs, a positive relationship between IBA and sleep was detected (*F*_1,26_ = 26.31, *p* < 0.001) (figure 7*b*).

**Figure 8.**
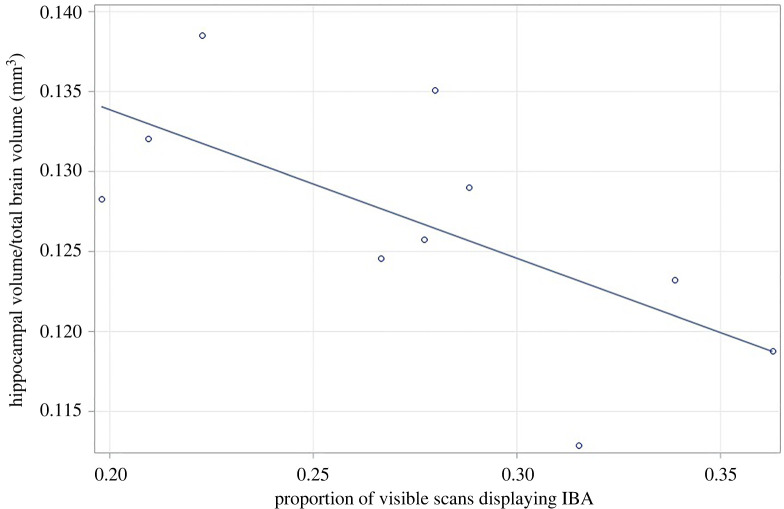
Relationship between proportion of visible observations where mice displayed IBA, and hippocampal volume, for conventionally housed C57 mice. Hippocampal volume corrected for total brain volume values are presented here as hippocampal volume/total brain volume for ease of interpretation, although analyses used hippocampal volume and included total brain volume as a covariate.

When assessing the effects of housing on hippocampal volume, there were no significant main or interactive effects of Housing. Assessing the relationship between IBA and hippocampal volume detected a significant IBA*Housing*Strain interaction (*F*_1,7.2_ = 10.17, *p* = 0.015), which revealed a significant negative relationship between IBA and hippocampal volume for conventionally housed C57s (*F*_1,7_ = 20.98, *p* = 0.003) (figure 8). The relationship between IBA and hippocampal volume was also negative, for C57s from well-resourced cages (*F*_1,1_ = 1.5737, *p* = 0.299) and conventionally housed DBAs (*F*_1,1_ = 5.033, *p* = 0.154), although non-significant. For DBAs from well-resourced cages, there was a non-significant relationship between IBA and hippocampal volume in the opposite direction, with hippocampal volume increasing with levels of IBA (*F*_1,1_ = 3.628, *p* = 0.153).

### 3.2. Experiment 2: refining the phenotyping of inactive-but-awake behaviour

#### 3.2.1. Cohort 1

**Figure 9.**
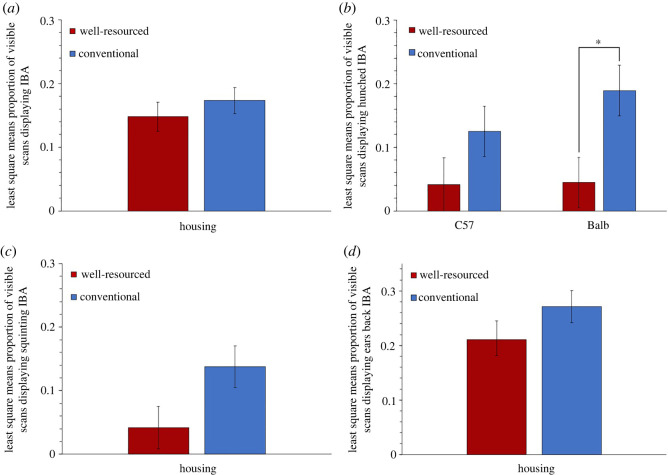
Housing effects in Experiment 2 (Cohort 1) on proportion of visible scans involving each form of IBA. Least-square means ± s.e. are presented for (*a*) IBA, (*b*) IBA with hunching, (*c*) IBA with squinting and (*d*) IBA with ears back (* = *p* ≤ 0.05).

**Figure 10.**
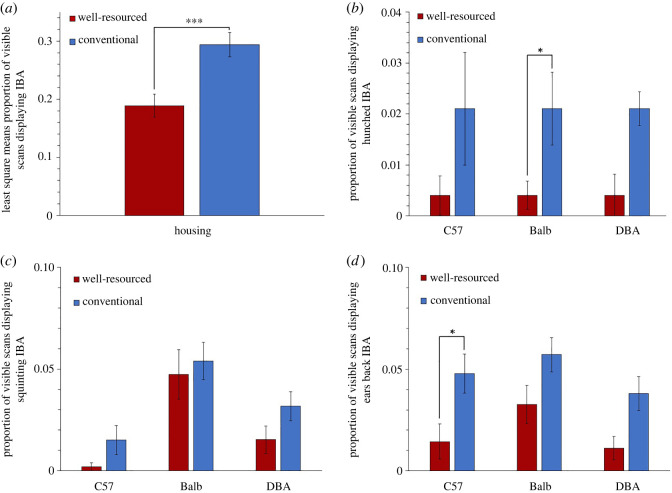
Housing effects in Experiment 2 (Cohort 2) on proportion of visible scans involving each form of IBA. Least square means ± standard error are presented for (*a*) IBA, and raw data means ± standard error are presented for (*b*) IBA with hunching, (*c*) IBA with squinting and (*d*) IBA with ears back (* = *p* ≤ 0.05, ** = *p* ≤ 0.01, *** = *p* ≤ 0.001).

In Cohort 1, when housing effects on IBA were assessed, we again found higher levels in conventional housing than well-resourced housing (all forms of the behaviour being included as in Experiment 1). However, the effect was non-significant, despite a medium effect size (*F*_1,12_ = 0.68, *p* = 0.427, Cohen's *d* *=* 0.336) (figure 9*a*), probably due to the small sample size of this pilot. By contrast, effect size comparisons for specific forms of IBA, in terms of posture and facial expression, as summarized in table 4, revealed some stronger housing effects. Note that all bouts of IBA with ears back were combined (pooling Score 1 and Score 2) since very few instances of ears severely pulled back were observed. The same was true for eye squinting. When only ‘IBA with hunching' was assessed, a trend for the effect of Strain*Housing was detected (*F*_1,8_ = 4.30, *p* = 0.072). Splitting by strain revealed a significant simple effect of Housing with a large effect size in Balbs (*t* = 2.58, d.f. = 8, *p* = 0.033, Cohen's *d* = 1.433), and in C57s, the Housing effect remained non-significant but the effect size generated was also large (*t* = 1.46, d.f. = 8, *p* = 0.183, Cohen's *d* = 0.886) (figure 9*b*). Models including only ‘IBA with squinting’ now detected a trend for the effect of Housing, and the effect size was increased to large (*F*_1,12_ = 4.44, *p* = 0.057, Cohen's *d* *=* 0.864) (figure 9*c*). By contrast, when only ‘IBA with ears back’ was assessed, the effect of Housing remained non-significant and the effect size remained medium (*F*_1,12_ = 1.78, *p* = 0.207, Cohen's *d* *=* 0.548) (figure 9*d* and figure 10).

**Table 4.** Summary of results for Experiment 2 (Cohort 1). Effect sizes are reported for comparison between each form of IBA.

IBA category	strain	housing	least-square mean	s.e.	*N*	Cohen's *d* for housing effect	effect size interpretation	*p*-value for housing effect
IBA	all mice	well-resourced	0.148	0.023	11	0.3364	medium	0.427
		conventional	0.173	0.021	13			
IBA with squinting	all mice	well-resourced	1.049	0.012	11	0.8636	large	0.057
		conventional	1.083	0.011	13			
IBA with hunching	all mice	well-resourced	0.043	0.039	11	0.8509	large	0.059
		conventional	0.157	0.038	13			
	C57s	well-resourced	0.041	0.042	4	0.8862	large	0.183
		conventional	0.125	0.039	7			
	Balbs	well-resourced	0.045	0.039	7	1.4332	large	0.033
		conventional	0.189	0.04	6			
IBA with ears back	all mice	well-resourced	0.211	0.034	11	0.5477	medium	0.207
		conventional	0.271	0.029	13			

#### 3.2.2. Cohort 2

When investigating housing effect on IBA in Cohort 2 mice, the model detected a significant main effect of Housing (*F*_1, 43.74_ = 13.29, *p* < 0.001, Cohen's *d* *=* 0.667), revealing higher levels of IBA in conventional housing than well-resourced housing. Because models investigating specific sub-forms of IBA did not meet parametric assumptions, Wilcoxon ranked sum tests were run for IBA, ‘IBA with hunching', ‘IBA with squinting’, ‘IBA with ears back’, and these were then used to compare housing effect sizes (table 5). As summarized in table 5, the Wilcoxon ranked sum test only detected a trend for a housing effect on IBA. Investigating specific forms of the behaviour in this cohort revealed that only hunched IBA in Balb mice generated a larger housing effect size, which now became significant. Interestingly, for DBA mice whose levels of IBA did not differ significantly between housing treatments, and whose levels of total IBA were slightly higher in animals from well-resourced cages, assessing only hunched IBA also reduced the magnitude of this negative effect size.

**Table 5.** Summary of results for Experiment 2 (Cohort 2). Effect sizes are reported for comparison between each form of IBA. Total IBA Wilcoxon ranked sum results are italicized for ease of comparison between categories. Levels of IBA are higher in well-resourced than conventional housing for all groups unless effect size is negative and interpretation is in brackets.

IBA type	strain	housing	*N*	mean	s.d.	*Z*	Wilcoxon *r*	effect size interpretation	*p*-value for housing effect
*IBA*	*Balb*	well-resourced	22	0.059	0.078	*2.010*	*0.307*	*medium*	*0.051*
		conventional	21	0.082	0.049				
IBA with hunching	Balb	well-resourced	22	0.004	0.013	2.298	0.350	medium	0.027
		conventional	21	0.021	0.033				
IBA with squinting	Balb	well-resourced	22	0.047	0.057	0.542	0.083	small	0.591
		conventional	21	0.054	0.042				
IBA with ears back	Balb	well-resourced	22	0.033	0.044	1.906	0.291	small	0.064
		conventional	21	0.057	0.039				
*IBA*	*C57*	well-resourced	19	0.093	0.083	*3.152*	*0.549*	*large*	*0.004*
		conventional	14	0.188	0.079				
IBA with hunching	C57	well-resourced	19	0.008	0.017	1.997	0.348	medium	0.054
		conventional	14	0.035	0.041				
IBA with squinting	C57	well-resourced	19	0.002	0.008	1.839	0.320	medium	0.075
		conventional	14	0.015	0.027				
IBA with ears back	C57	well-resourced	19	0.014	0.038	3.018	0.525	large	0.005
		conventional	14	0.048	0.036				
*IBA*	*DBA*	well-resourced	22	0.028	0.047	*−1.529*	*−0.231*	*(small)*	*0.134*
		conventional	22	0.046	0.047				
IBA with hunching	DBA	well-resourced	22	0.004	0.019	−0.510	−0.077	(small)	0.613
		conventional	22	0.005	0.016				
IBA with squinting	DBA	well-resourced	22	0.015	0.032	−1.898	−0.286	(small)	0.065
		conventional	22	0.032	0.033				
IBA with ears back	DBA	well-resourced	22	0.011	0.027	−2.648	−0.399	(medium)	0.011
		conventional	22	0.038	0.039				

## 4. Discussion

Our aim was to investigate IBA in laboratory mice: a specific form of waking inactivity with the potential to be a simple cageside welfare assessment tool, and perhaps even a sign of depression-like states. Experiment 1 tested for the presence of several depression-like attributes in mice displaying IBA, and Experiment 2 sought to identify physical postures or expressions that could differentiate this form of inactivity from normal resting. The shared aim of both experiments was improving the value of home cage inactivity as a cageside metric to evaluate mouse welfare.

The depression hypothesis tested in Experiment 1 predicted that (i) levels of IBA would be higher in conventional housing than well-resourced housing and that high IBA mice would (ii) show more immobility during Forced Swim Tests, (iii) show unusually high or low levels of sleep (since both insomnia and hypersomnia are characteristic of depression), (iv) have unusually high or low BMIs (since both weight loss and weight gain are characteristic of depression), and (v) have relatively small hippocampi. All predictions were fully or partially supported. Thus first, levels of IBA were higher in conventional than well-resourced cages for the two strains prone to inactivity: Balbs and C57s. The absence of similar housing effects in DBA mice was not surprising as this replicates past findings [14], with these individuals demonstrating low levels of IBA, and instead showing active stereotypic behaviour in response to conventional cages. Turning to behavioural correlates of IBA, group differences in Forced Swim Test performance mirrored housing and strain differences in home cage IBA. Here, conventionally housed mice spent more time floating immobile in C57 and Balb strains, but effects were not detected for DBAs who performed very little floating. In terms of individual differences, there was also a positive relationship between IBA and Forced Swim Test immobility, at least for conventionally housed mice. The same did not hold for animals from well-resourced cages, but this was likely a floor effect caused by their very low levels of both IBA and Forced Swim Test immobility (probably a result of considerably improved well-resourced cages in this experiment compared with past work, i.e. with increased floor space and many more enrichments [14]). Assessing sleep patterns during the active phase of the diurnal cycle similarly revealed strain and housing effects that at least partially mapped onto those for IBA. Thus for C57 and Balb strains, conventional caging increased the time spent sleeping. C57 and Balb mice also slept more than DBAs, though C57 mice slept less than Balbs (despite their higher levels of IBA). Turning to individual differences, mice spending more time IBA also slept more in DBA mice, and in conventionally housed Balb mice, but there was a lack of apparent relationship between IBA and sleep in C57 mice. Future studies might explore this relationship further using 24 h data for sleep, and perhaps EEG as well as behavioural measures.

IBA mice showed some of the predicted physical characteristics of depression too. Strain differences in BMI reflected strain differences in IBA, with C57s having the highest and DBAs the lowest. However, housing effects here did not mirror those on IBA: BMIs of conventional and well-resourced mice did not differ within the C57s, and for the other two strains, conventionally housed Balb and DBA mice had *lower* BMIs than well-resourced conspecifics (an effect probably driven by the high levels of stereotypic behaviour in conventionally housed mice of these strains). Nonetheless, individual mice spending more time IBA had greater BMIs than their more active conspecifics, an effect that held across all three strains. Finally, we assessed hippocampal volumes, albeit only for two strains and with a rather low sample size. Unexpectedly, given a large body of previous work investigating housing effects on this measure (e.g. [76–78]), our well-resourced mice did not have larger hippocampi than conventionally housed peers. However, we suspect this was a Type II error caused by low power, and now plan to replicate this work using more subjects. Younger animals, whose brains are embedded before sectioning, will also be used to avoid sample loss due to breakage during processing. However, investigating the relationship with IBA revealed that in conventionally housed C57 mice (the subgroup with the highest IBA and also the most brain samples analysed), individuals who had had the highest levels of IBA also had the smallest hippocampi relative to brain size.

Taken together, across three strains, this work consistently links IBA with two potential signs of depression: helplessness in Forced Swim Tests and weight gain. IBA additionally predicted increased sleep in two strains (DBAs and conventionally housed Balbs): consistent with the depressive criterion of hypersomnia, which in humans, commonly manifests as increased daytime sleep [26]. This evidence is not sufficient to confirm depression in IBA mice, since it is the co-occurrence of five or more specific diagnostic criteria that is crucial [6,26]. However, it does indicate the value of now testing for co-occurring additional signs of depression, such as cognitive deficits, anhedonia, and low mood as inferred from ‘pessimism’ in judgement bias tasks (see MacLellan *et al*. [6] and Resasco *et al*. [72]). Furthermore, the sleep and body weight data suggest that if the depression hypothesis is supported, mice may experience the ‘atypical’ subtype (as opposed to melancholic; [26,79]). This in turn predicts testable differences in two biomarkers: low levels of circulating corticosteroids [79] and high levels of proinflammatory cytokines [80]. Such work could also explore whether body fat increases individual predispositions to IBA via inflammation (adipose being inflammatory [80]), since our results unexpectedly showed that IBA could not be the cause of increased BMI (conventionally housed mice paradoxically showing higher IBA but being no fatter than well-resourced conspecifics).

The one biomarker we looked at here further supported the depression hypothesis, at least for conventionally housed C57 mice: individuals with high IBA had reduced hippocampal volumes. While reduced hippocampal volumes are common in human patients with clinical depression [45], they are not specific to this condition (e.g. also being characteristic of post-traumatic stress disorder [81]). Nonetheless, this link also confirms the welfare significance of IBA (even if these mice are not depressed), since chronic stress and psychological trauma reliably reduce hippocampal volumes across diverse species [71]. Similarly, the link between IBA and Forced Swim Test immobility provides support for the hypothesis that IBA reflects depression, but does not rule out alternative underlying conditions. This is because helplessness—if that is indeed what this test is detecting—is elevated not only in depression, but in many individuals experiencing the loss of control [36]. Still, Forced Swim Tests results shown here add to the evidence that IBA indicates poor welfare. This is because Forced Swim Test immobility in rodents can be increased by exposure to various aversive experiences like repeated social defeat [82] and unpredictable environmental stressors (combining tilted cages, wet bedding, altered light cycles, white noise etc.) [83]. Thus, whether or not IBA reflects depression, these data further validate it as an indicator of poor welfare that could be useful for cageside assessment. Future research should now investigate IBA's welfare significance and generalizability further. This could involve assessing this behaviour in males (since only investigated in females to date), in mice from diverse strains and in mice kept in same-strain housing. Further, IBA should also be assessed in laboratory rats, who like C57 mice, are prone to obesity in conventional cages [84], and typically show rather little stereotypic behaviour. Investigating IBA's sensitivity to acute challenges (e.g. ear-notching), as well as to additional chronic ones (e.g. repeated tail-handling, or social isolation) is also now warranted. Finally, exploring IBA's relationship to non-depressive negative states such as boredom, pain or anxiety is needed before such underlying causes can be ruled out.

With cageside welfare assessments in mind, we then aimed to identify characteristics of IBA that could help refine its phenotype and specificity as an indicator of negative affect. We did this by measuring which forms of IBA were most increased by conventional housing. Experiment 2, the first ever investigation of mouse facial and postural changes occurring in-cage, found effects that appeared to vary with strain and cohort. For Balb subjects, housing effect sizes for IBA were consistently larger if only IBA involving hunched postures was scored. This suggests that for this strain, conventional cages induce hunched forms of inactivity resembling those of mice who are sick or socially defeated. ‘Hunched IBA’ is thus a more accurate welfare indicator for Balbs than pooling all forms of their IBA together. In one of our two cohorts (Cohort 1), the other two strains showed similar effects for hunched postures, suggesting it might have broader promise. However, this did not hold for Cohort 2 (C57s and DBAs in conventional cages not being prone to hunching). Furthermore, in all three strains in Cohort 1, the elevated IBA in conventional cages involved eye squinting, thus resembling expressions seen in mice experiencing pain or fear [54–56]. However, again such patterns were not replicated in the second cohort, leaving their importance uncertain. For C57s in particular, the strain most prone to IBA, we therefore need further work to refine the phenotype of this behaviour and enhance its accuracy as a welfare indicator. This could involve looking at combinations of facial expressions and postural changes, attributes we could not assess (like subtle cheek bulges included in the Mouse Grimace Scale), IBA bout durations, or the interruptibility of IBA (cf. the low responsiveness of ‘withdrawn’ horses [11]). This would parallel similar, fruitful, investigations of the physical characteristics of welfare-significant inactivity by Fureix *et al.* on horses [10,11], Hennessy *et al.* on laboratory macaques [7,8], Hintze *et al.* on cattle [85] and Meagher *et al.* on mink [48,86]. The resulting refined ethograms could then enhance the validity and practical value of mouse IBA as a welfare indicator: important as these animals are used extensively in research, and their welfare is attracting growing scrutiny [87].

## 5. Conclusion

Understanding and validating behavioural indicators of mouse welfare is essential for refining how they are housed, managed and treated in research. Most welfare studies and cageside assessment schemes focus on physical condition and active forms of abnormal behaviour. But, just as in other species, an unusual form of inactivity—‘IBA’ behaviour—is emerging as a new indicator of poor mouse welfare, one especially useful for strains that display little stereotypic behaviour. IBA is elevated by suboptimal housing, predicts ‘helplessness' in Forced Swim Tests and, at least in one strain, is associated with hippocampal volume loss. IBA also shows some depression-like attributes, although further research is imperative to definitively accept or reject the depression hypothesis, and to explore alternative explanations. Refining the phenotype of IBA is an ongoing challenge, but hunched postures appear to be a promising characteristic, and in Balb mice, only scoring IBA that involves such postures significantly improves the accuracy of IBA as a welfare indicator. The methods outlined here therefore present a promising approach for future work aiming to refine the phenotype of IBA, an important endeavour given the convergent evidence that IBA is a sign of poor welfare, and the heavy reliance on mice in research.

## Data Availability

The datasets supporting this article have been uploaded as part of the electronic supplementary material [89].
